# Chronic Liver Disease Associated Cholangiocarcinoma: Genomic Insights and Precision Therapeutic Strategies

**DOI:** 10.3390/cancers17183052

**Published:** 2025-09-18

**Authors:** Kyoko Oura, Asahiro Morishita, Mai Nakahara, Tomoko Tadokoro, Koji Fujita, Joji Tani, Tsutomu Masaki, Hideki Kobara

**Affiliations:** 1 Department of Gastroenterology and Neurology, Faculty of Medicine, Kagawa University, Kita-gun 761-0793, Japan; 2 Department of Gastroenterology, Kagawa Saiseikai Hospital, Takamatsu 761-8076, Japan

**Keywords:** cholangiocarcinoma, etiology, genomic alterations, molecular profiling, FGFR2 infusion, IDH1 mutation, ERBB2 amplification, target therapy, precision oncology, chronic liver disease

## Abstract

**Background**: Cholangiocarcinoma (CCA) is a highly heterogeneous malignancy arising from the biliary epithelium, with an increasing incidence and poor prognosis worldwide. Recent advances in next-generation sequencing have revealed a variety of genomic alterations―such as FGFR2 fusions, IDH1 mutations, and ERBB2 amplification―that may serve as therapeutic targets. However, the influence of underlying etiologic factors, including chronic liver and biliary diseases, on the molecular landscape of CCA remains unclear. **Objective**: This review aimed to synthesize the current knowledge on the genomic and molecular alterations of CCA in the context of diverse etiologic factors, including hepatitis B virus, hepatitis C virus, primary sclerosing cholangitis (PSC), primary biliary cholangitis, metabolic dysfunction-associated steatotic liver disease (MASLD), alcohol-related liver disease (ALD), and liver fluke infection. **Main findings**: Certain backgrounds, such as PSC and liver fluke infection, are associated with distinct molecular signatures (e.g., TP53, SMAD4, KRAS, and ERBB2 alterations), whereas others, such as MASLD or ALD, show limited and inconsistent genomic data. Targetable alterations―including FGFR2 fusions, IDH1 mutations, and ERBB2 amplification―are heterogeneously distributed across etiologies and anatomical subtypes. Molecular targeted therapies such as FGFR and IDH1 inhibitors have shown clinical benefits in selected patients. **Conclusions**: A better understanding of how chronic liver and biliary diseases shape the genomic landscape of CCA will inform the development of personalized treatments, surveillance strategies, and preventive approaches. Large-scale etiology-stratified genomic studies integrating multiomics and real-world clinical data are urgently needed to advance precision oncology in CCA.

## Simple Summary

Cholangiocarcinoma (CCA) is a rare but aggressive biliary tract cancer that is often diagnosed at an advanced stage. Recently, certain genetic changes that play critical roles in the development of CCA have been identified, leading to the development of new targeted therapies. However, how different causes of liver and bile duct diseases, such as hepatitis viruses, cholestatic liver diseases, and parasitic infections like liver flukes, affect the genetic features of CCA is still not fully understood. In this review, we summarize the current knowledge on how various underlying conditions influence the molecular characteristics of CCA and its response to treatment. We also highlight the latest advances in precision medicine, including the development of therapies targeting specific gene mutations. Understanding these relationships may help improve the diagnosis, treatment, and prevention of CCA.

## 1. Introduction

Cholangiocarcinoma (CCA) comprises a heterogeneous group of malignancies arising from the bile duct epithelium and accounts for approximately 15% of primary liver cancers [1]. According to the WHO 2019 tumor classification, CCA is primarily categorized based on anatomical location into intrahepatic CCA (iCCA) and extrahepatic CCA (eCCA). iCCA is further subdivided into small-duct type and large-duct type, while eCCA is classified into perihilar and distal types according to the site of origin within the extrahepatic biliary tree [2]. In addition, CCA can be classified according to tumor growth patterns into three distinct forms: mass-forming, periductal infiltrating, and intraductal types. This classification framework provides a standardized basis for diagnosis, molecular characterization, and treatment strategies and is now widely adopted in clinical practice worldwide.

Although its global incidence remains relatively low (fewer than 2 cases per 100,000 people annually), it shows marked geographic variation, with a notably higher incidence in parts of East Asia [3]. In Southeast Asia, the endemic presence of liver flukes―notably *Opisthorchis viverrini* and *Clonorchis sinensis*―substantially contributes to the disease burden [4], whereas in regions such as Japan, Korea, and Western countries, CCA is more commonly linked to chronic liver and biliary diseases, including hepatitis B virus (HBV) and hepatitis C virus (HCV) infections [5,6], primary sclerosing cholangitis (PSC) [7,8,9], metabolic dysfunction-associated steatotic liver disease (MASLD) [10,11], and alcohol-related liver disease (ALD) [12]. The incidence of CCA has been reported to be increasing in several countries and is thought to be influenced by improvements in diagnostic imaging and the increasing prevalence of risk factors, such as metabolic syndrome and chronic viral hepatitis [13,14,15].

CCA is frequently diagnosed at an advanced stage, primarily because of its asymptomatic nature in the early phases and the anatomical complexity of the biliary tree, which hampers early detection. As a result, curative surgical options are often not feasible at the time of diagnosis, and the prognosis of unresectable cases remains poor, with median survival times of around 12 months under standard systemic therapy [16,17]. For more than a decade, the combination of gemcitabine and cisplatin has served as the standard first-line chemotherapy regimen for advanced CCA, providing modest survival benefit [18]. More recently, the addition of the immune checkpoint inhibitor durvalumab to this backbone has demonstrated additional clinical benefits, representing a step forward in systemic therapy for these patients [17]. Nevertheless, the efficacy of immune checkpoint inhibitors in CCA remains limited, especially when compared to so-called immune “hot” tumors such as melanoma or non-small cell lung cancer. This highlights the pressing need for novel therapeutic approaches beyond immunotherapy.

The advent of next-generation sequencing (NGS) has substantially advanced our understanding of CCA molecular heterogeneity. Through comprehensive genomic analyses, various actionable mutations have been identified, including fibroblast growth factor receptor 2 (FGFR2) fusions [19,20], isocitrate dehydrogenase 1 (IDH1) and IDH2 mutations [21,22], BRCA1-associated protein 1 (BAP1) loss [19], and other alterations in chromatin remodeling genes. Importantly, the prevalence and spectrum of these molecular alterations vary according to the underlying etiology and anatomical subtype of CCA, reflecting the complex interplay between environmental, infectious, immune-mediated, and metabolic factors in carcinogenesis. Despite the significant progress in mapping the genomic landscape of CCA, most reviews have focused on specific anatomical subtypes, isolated molecular targets, or single etiological factors. Consequently, an integrative understanding of the diversity of chronic liver and biliary conditions such as HBV, HCV, PSC, primary biliary cholangitis (PBC), MASLD, and ALD is needed to clarify how these backgrounds influence the genomic and molecular characteristics of CCA. While strong associations have been observed in certain etiologies (e.g., PSC and liver fluke infection), comprehensive data across most backgrounds are limited or inconclusive.

In this review, we aim to bridge this critical gap by systematically summarizing the current knowledge on the genomic and molecular landscape of CCA in the context of its diverse etiologies. In doing so, we not only highlight known and emerging associations but also identify areas where evidence is scarce, thereby laying a foundation for future investigations in precision oncology for CCA. This narrative review is based on a comprehensive survey of the relevant literature, drawing on recently published peer-reviewed articles, landmark studies, and authoritative reviews in the field, with particular attention to advances reported in the past decade.

## 2. Chronic Liver and Biliary Diseases as Etiologic Factors

**Figure 1:**
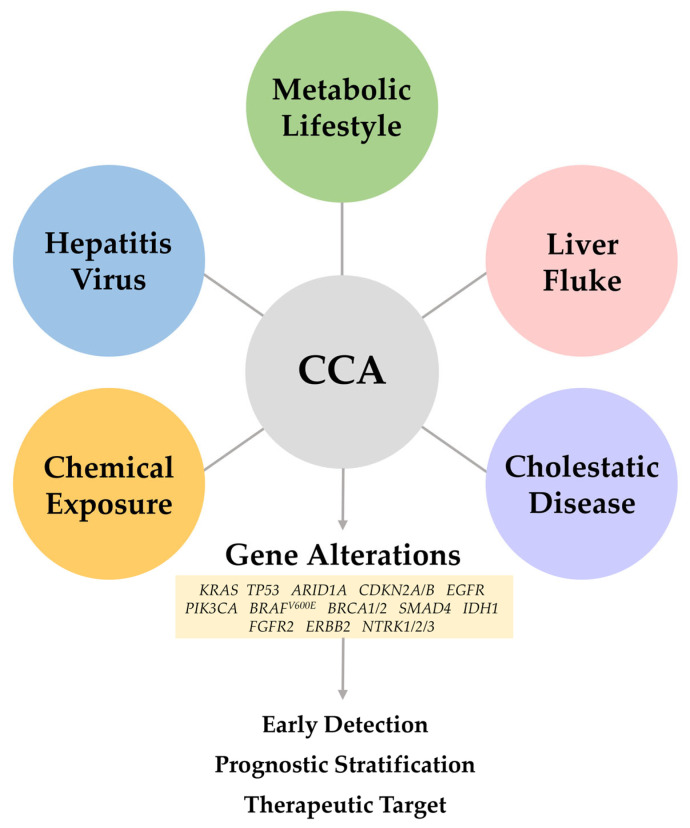
Overview of the etiologic factors contributing to cholangiocarcinoma (CCA) and their associated genomic alterations. Chronic viral infections, metabolic and lifestyle-related disorders, liver fluke infections, cholestatic diseases, and chemical exposures shape the molecular heterogeneity of CCA. Cholestatic disease includes primary sclerosing cholangitis, hepatolithiasis, and Caroli disease. This diversity in etiology underpins the development of distinct mutational landscapes, which may offer early detection and surveillance, prognostic stratification, and identification of potential targets for precision therapies. CCA: cholangiocarcinoma, KRAS: Kirsten rat sarcoma viral oncogene homolog, TP53: tumor protein p53, ARD1A: AT-rich interactive domain 1A, CDKN2A/B: cyclin-dependent kinase inhibitor 2A/B, EGFR: epidermal growth factor receptor, PIK3CA: phosphatidylinositol-4,5-bisphosphate 3-kinase catalytic subunit alpha, BRAF: B-Raf proto-oncogene, serine/threonine kinas, BRCA1/2: breast cancer gene 1/2, SMAD4: SMAD family member 4, IDH1: isocitrate dehydrogenase 1, FGFR2: fibroblast growth factor receptor 2, ERBB2: Erb-B2 receptor tyrosine kinase 2, NTRK1/2/3: neurotrophic tyrosine receptor kinase 1/2/3.

Figure 1 provides an overview of the etiologic factors associated with CCA. The etiological and molecular associations depicted are primarily derived from clinical observations, genomic analyses, and epidemiological data. However, establishing causal links between specific risk factors and the activation of oncogenic signaling pathways requires further mechanistic validation. Importantly, various genes associated with these etiologies may hold significant clinical value in early detection, prognostic stratification, and the development of targeted therapeutic strategies.

### 2.1. Environmental and Cholestatic Risk Factors for CCA

Risk factors for CCA development include chemical exposure, congenital or acquired biliary obstruction leading to cholangitis, chronic hepatic inflammation, chronic biliary inflammation, and cirrhosis. Occupational and lifestyle-related exposures may also contribute. Exposure to certain chemical and environmental agents, such as 1,2-dichloropropane, dichloromethane [22], and asbestos [23], has been associated with the development of CCA. These chemicals and their metabolites contribute to cholangiocarcinogenesis not only through the induction of DNA damage, mutagenesis, and oxidative stress but also by promoting persistent cholangitis and pathological alterations of the bile duct epithelium, which are frequently observed in CCA [24]. Accordingly, exposure to these substances is regarded as a key molecular driver of CCA.

Furthermore, infection with liver flukes, such as *Clonorchis sinensis* and *Opisthorchis viverrini* through the consumption of raw or undercooked fish, is a well-established cause of CCA in Asian regions, including South Korea and China [25]. Other liver flukes, such as *Fasciola hepatica* and *Fasciola gigantica*, have not been directly proven to cause CCA but can lead to fascioliasis, an animal-borne infection that may contribute to chronic bile stasis and thereby potentially act as a risk factor [26].

In the context of chronic cholestasis, a direct association between hemolytic anemia, such as sickle cell anemia or glucose-6-phosphate dehydrogenase deficiency, and the development of CCA has not been established. However, given that these conditions can cause bilirubin stone formation, which in turn may lead to chronic intrahepatic cholestasis, their potential contribution to the risk warrants attention [27]. Bilirubin stones are recognized as a contributor to chronic intrahepatic cholestasis and may play a key role in the pathogenesis of CCA.

### 2.2. Risks of Developing CCA from Hepatitis Viruses

Hepatitis viruses, including HBV and HCV, are recognized risk factors for CCA as well as for hepatocellular carcinoma (HCC), non-Hodgkin’s lymphoma, multiple myeloma, and thyroid cancer. According to a meta-analysis evaluating the risk of CCA, hepatic viruses are associated with an increased risk of CCA, particularly iCCA, with a significant increase in risk demonstrated by an odds ratio of 3.96 for HBV infection and 2.90 for HCV infection [28]. A meta-analysis including 29 case–control studies and 9 cohort studies found that HBV infection is associated with significantly increased risk of CCA, with odds ratios of 1.75 for overall CCA, 1.49 for eCCA, and 2.46 for iCCA [5]. Meanwhile, this study also demonstrated that HCV infection is associated with a significantly increased risk of CCA, with pooled odds ratios of 1.45 for overall CCA, 2.00 for eCCA, and 2.81 for iCCA [5].

According to a study using tumor tissue from patients with iCCA who underwent liver resection, 40.7% of the tissue was positive for HBs antigen in immunohistochemical staining. Furthermore, patients with HBV-positive iCCA had significantly shorter disease-free survival than those with HBV-negative iCCA. Interestingly, the HBV antigen was detected only in fresh iCCA tissues and organoids, but not in bile duct epithelial cells in the portal area. This localization of HBV suggests that HBV-associated iCCA may originate from hepatocytes rather than bile duct epithelial cells [29]. According to another retrospective study, HBV-positive iCCA differed from HBV-negative iCCA in pathological features, such as tumor density uniformity, vascular encasement invasion, arterial phase, overall reinforcement pattern, biliary dilatation, and peritumoral bile duct stones [30]. However, there was no difference in the postoperative progression-free survival (PFS). There are discrepancies in the results of studies on the prognosis of HBV-associated CCA, and further verification is necessary.

However, evidence regarding the risk of CCA associated with HCV infection, particularly the presence or absence of a history of HCV treatment, is more abundant in Asia. In a large-scale Asian cohort study of 11,892,067 individuals, the cumulative incidence of eCCA was 0.088% in HCV-treated patients, 0.095% in untreated patients, and 0.048% in HCV-uninfected individuals [31]. Although the lowest rate of eCCA was observed in the uninfected group, there was no significant difference between the treated and untreated HCV groups. A recent report from Japan evaluated the prognosis of patients with iCCA who underwent curative resection and showed no significant differences in recurrence-free survival (RFS) or overall survival (OS) between HCV-infected and uninfected patients [32]. Similarly, no differences in outcomes were observed based on the presence or absence of a sustained virological response (SVR). However, the multivariate Cox regression analysis identified non-SVR as an independent poor prognostic factor for RFS. When these findings are considered along with those of previous meta-analyses [5], HCV infection appears to be more strongly associated with the risk of iCCA than with eCCA. Although viral clearance through HCV treatment may influence the development or prognosis of CCA, the specific antiviral regimen and treatment duration are likely to affect the degree of intrahepatic inflammation and subsequent CCA development. Further systematic investigations are required to clarify these associations.

### 2.3. Risks of Developing CCA with Chronic Cholestatic Liver Diseases

Chronic cholestatic liver diseases, exemplified by PSC, contribute to the development of CCA through persistent cholangitis and prolonged bile stasis. PSC is a rare, idiopathic biliary liver disease characterized by chronic inflammation of the major extrahepatic and intrahepatic bile ducts and is one of the most well-known risk factors for CCA. The American Association for the Study of Liver Diseases Practice Guidelines also state that CCA can develop at any time during the course of PSC, with an incidence rate of 1–1.5% per year, and that the cumulative risks of CCA 10, 20, and 30 years after PSC onset are 6%, 14%, and 20%, respectively [8]. Compared with the general population, the risk of CCA is 160–400 times higher. The incidence of CCA in patients with PSC varies from 4% to 36%, with a particularly high risk in the early stages after PSC diagnosis; 30% to 50% of CCA cases are diagnosed within the first year [9]. A recent systematic review and meta-analysis of approximately 26,000 cases showed an incidence rate of 9.31 for CCA over a total follow-up period of 221,258.1 person-years, which was higher than the incidence rate of 1.73 for HCC [33]. Additionally, the incidence rate was higher in patients with concomitant inflammatory bowel disease.

However, there is insufficient evidence to indicate an increased risk of CCA alone in patients with PBC and underlying bile duct disorders caused by chronic cholangitis or fibrosis. In a Swedish cohort study of 3052 PBC patients followed up for 10 years, the overall cancer incidence rate was 14.3%, compared to 11.8% in the matched control group, with significantly higher risks of gastrointestinal cancer, lung cancer, and lymphoma [34]. However, in a meta-analysis of 16,300 patients with PBC, although the overall cancer risk increased by 1.55-fold, no clear association with other specific cancers, including CCA, was established [35].

Hepatolithiasis is also a recognized risk factor for CCA, accounting for 3–10% of all cases [36]. Several clinical features have been associated with an increased risk of CCA development in patients with hepatolithiasis. These include age over 40 years, a prolonged history of hepatolithiasis exceeding 10 years, previous infection with HBV, a history of smoking, a family history of cancer, elevated alkaline phosphatase, high levels of a tumor marker, low serum albumin, and biliary abnormalities such as ductal strictures, focal or global hepatic atrophy, and bilateral stone disease [36,37]. Additionally, a history of gastrectomy or choledochoenterostomy has also been associated with a higher risk. Hepatolithiasis-associated CCA is typically classified into two types: concomitant cases that are diagnosed at the same time as hepatolithiasis, and subsequent cases that develop after treatment of hepatolithiasis. The incidence of concomitant CCA ranges from 5 to 12%, while subsequent cases have been reported in up to 10% of patients [38]. These pathological conditions lead to repeated damage and regeneration of epithelial tissue, forming genetic alterations that promote CCA development [1].

Furthermore, congenital biliary tract disorders, such as Caroli disease and congenital hepatic fibrosis, are also recognized as risk factors for iCCA. Caroli disease, in particular, is a rare congenital liver disorder characterized by segmental cystic dilatation of the intrahepatic bile ducts, resulting from incomplete remodeling of the ductal plate during embryogenesis [39]. These abnormalities lead to chronic cholestasis and bile stasis, promoting chronic inflammation and bile duct dysplasia, which may eventually progress to malignancy. The estimated rate of malignant transformation in these conditions ranges from 7% to 16% [40], highlighting the importance of early detection and appropriate management.

### 2.4. Risks of Developing CCA with Other Liver Diseases

MASLD, formerly termed nonalcoholic fatty liver disease, is strongly associated with cirrhosis and HCC [41]. However, its association with CCA is not as clear as that with HCC. Obesity, which is commonly observed in patients with MASLD, is also a known risk factor for CCA [42,43] and may provide evidence of a potential causal relationship between MASLD and CCA. In a study evaluating the prevalence of MASLD in tissues surrounding the CCA that had undergone resection, the prevalence of MASLD was 6.2% in the study group, whereas 22.5% of patients with CCA without known classical risk factors were found to have MASLD [44]. According to a meta-analysis, MASLD increases the risk of developing common bile duct cancer by an odds ratio of 1.88 and iCCA by an odds ratio of 2.19, but has no significant effect on the development of eCCA [11]. Another recent large-scale cohort study using Swedish data showed that among 11,940 exposed patients with MASLD followed for 5 years, 11 (0.1%) developed CCA, and three (0.03%) were diagnosed with iCCA. However, the incidence of CCA did not differ significantly between the MASLD and control groups [45]. The fact that studies provide different perspectives on the impact of MASLD on increased CCA risk suggests that MASLD itself does not directly influence CCA risk, but risk may be mediated through obesity and metabolic disorders.

ALD may promote the development of HCC and CCA through chronic inflammation and oxidative stress. A meta-analysis of seven studies involving 413,483 healthy controls and 8962 patients with CCA showed that patients with ALD had a 3.92-fold higher risk of CCA than the normal control group [12]. Interestingly, a subgroup analysis revealed that ALD was associated with an increased risk of iCCA rather than eCCA; further stratification by geographical region showed that a positive association was observed in Western European countries rather than in Eastern European countries. A large cohort study of patients with cirrhosis caused by ALD showed that the 5-year cumulative cancer risk for iCCA was 0.07% in England and 0.05% in Denmark, with the 10-year risk remaining at similar levels, providing further evidence to support these findings [46].

## 3. Common Genomic and Molecular Alterations in CCA

Building on the etiological heterogeneity discussed above, we explored how these distinct backgrounds shape the genomic and molecular profiles of CCA, thereby influencing its biological behavior and therapeutic vulnerabilities. The molecular heterogeneity of CCA presents challenges to uniform treatment strategies. The European Society for Medical Oncology guidelines recommend routine genomic profiling before initiating treatment and prioritizing targeted therapy if treatable alterations are present [47]. Indeed, genomic testing and targeted therapy increasingly play central roles in CCA management. Common genomic alterations are observed among anatomical subtypes in some cases of CCA. However, the frequency of these alterations differs significantly between iCCA and eCCA, suggesting that tumor location and the associated microenvironment may play important roles in the progression of CCA. Alterations frequently reported in both iCCA and eCCA include Kirsten rat sarcoma viral oncogene homolog (KRAS), tumor protein 53 (TP53), AT-rich interactive domain 1A (ARID1A), and cyclin-dependent kinase inhibitor 2A/B (CDKN2A/B), while IDH1/2, epithelial growth factor receptor (EGFR), and FGFR2 alterations are reported at high rates in iCCA but are rare in eCCA. Table 1 lists the gene alterations and their frequencies.

### 3.1. Common Genetic Alterations Seen in CCA

KRAS plays a central role in mediating upstream to downstream signal transduction and regulates cell differentiation and proliferation by switching between an active GTP-bound form and an inactive GDP-bound form [73]. When ligands bind to receptor tyrosine kinases such as EGFR or anaplastic lymphoma kinase (ALK) located on the cell membrane, KRAS is activated and transmits the signal to downstream serine/threonine kinases such as mitogen-activated protein kinase (MAPK)/extracellular signal-regulated kinase (ERK) or phosphoinositide 3-kinase (PI3K)/protein kinase B (AKT), leading to cell proliferation or inhibition of apoptosis [74]. Active KRAS hydrolyzes GTP through its intrinsic GTPase activity and the GTPase-activating protein, converting it back to its inactive form. KRAS mutations are relatively common in CCA, with a reported frequency of 24–27% in iCCA [49,50] and 37–46% in eCCA [48,49,51,52], suggesting a slight tendency toward higher prevalence in eCCA. Among KRAS mutations, those at G12D, G12V, and Q61H are particularly common, and alterations at G12/G13 are strongly associated with poor prognosis [75]. KRAS mutations can also be detected using liquid biopsy with circulating cell-free DNA, and the concordance rate with tissue samples is relatively high, making it a promising noninvasive method for prognostic evaluation.

TP53 is a tumor suppressor gene that responds to various cellular stresses such as DNA damage, oncogene expression, and hypoxia, and performs a wide range of functions that suppress cancer, including DNA repair, cell cycle arrest, apoptosis induction, aging, metabolic regulation, and immune responses [76,77]. It is one of the genes with the most frequent alterations in human cancers. These alterations have a negative dominant effect that impairs the function of normal p53 and a gain-of-function that acquires a new cancer-promoting function [78,79]. The frequency of TP53 mutations is generally high in CCA but has been reported to be 20–27% in iCCA [50,53] and 35–68% in eCCA [48,50,52]. Although there are some variations, previous reports have suggested that eCCA tends to occur at a higher frequency.

ARID1A is a chromatin remodeling factor that regulates gene expression by adjusting chromosome structure. It also plays a role in transcriptional control, DNA repair, and cell cycle regulation [80]. In chromatin-dependent cellular processes, the Switch/Sucrose non-fermentation complex, a member of the chromatin remodeling complex in mammals, alters DNA accessibility via nucleosome movement, expulsion, or exchange. Among these alterations, ARID1A was the most frequent [81]. As a tumor suppressor, it has been reported to have a high frequency of loss-of-function mutations in various cancers, including ovarian and endometrial cancers. Its frequency in CCA has been reported to be 18–23% in iCCA [50,53] and 14% in eCCA [48]. ARID1A mutations are particularly common in iCCA and are associated with the prognosis and immune responses. A recent study has shown that loss of ARID1A in iCCA cells leads to upregulation of stemness-related markers such as ALDH1A1 [82]. Mechanistically, ARID1A represses ALDH1A1 transcription by recruiting histone deacetylase 1 and reducing histone H3K27 acetylation at the ALDH1A1 promoter. These findings suggest that ARID1A loss not only promotes epigenetic dysregulation but also enhances cancer stemness, potentially contributing to tumor aggressiveness and poor prognosis. According to another study, low ARID1A expression is associated with poor prognosis in iCCA patients, suggesting it may serve as a potential prognostic biomarker candidate for iCCA [83]. Tumors harboring ARID1A mutations tend to exhibit high microsatellite instability or high tumor mutational burden, suggesting increased sensitivity to immune checkpoint inhibitors [84,85]. Furthermore, the co-occurrence of activating KRAS mutations and ARID1A deletions has been shown to synergistically accelerate cholangiocarcinoma development from cholangiocytes, especially under conditions of liver inflammation [86]. ARID1A loss induces uncontrolled cell proliferation and alters chromatin structure, contributing to malignant transformation. Mechanistically, this process involves suppression of the TGF-β/Smad4 tumor suppressor pathway, establishing an ARID1A-TGF-β-Smad4 axis as critical in preventing biliary carcinogenesis.

CDKN2A encodes p16INK4a and p14ARF, whereas CDKN2B encodes p15INK4b, which are major regulators of the G1/S cell cycle checkpoint via the CDK4/6–Rb and p53 signaling pathways [87,88]. CDKN2A/B gene mutations are known to be highly prevalent in pancreatic cancer [89], skin melanoma [90], and lung cancer [91]. These are also common gene alterations in CCA, with recent studies reporting that CDKN2A/B gene mutations are observed in 15–27% of iCCA cases [50,54] and 19% of eCCA cases [50,52]. Patients with advanced CCA and CDKN2A/B gene mutations have a shorter time from initial chemotherapy to disease progression and poorer overall survival [53]. Furthermore, in patients with FGFR2 fusion-positive iCCA, the CDKN2A/B deletion emerged after the development of acquired resistance to FGFR inhibitors [92], suggesting a potential mechanism underlying FGFR inhibitor resistance and supporting the association of this genetic alteration with poor prognosis.

EGFR, a member of the ERBB family of receptor tyrosine kinases, plays a pivotal role in regulating cellular proliferation, survival, and differentiation [93]. It is widely recognized for its oncogenic role in various human cancers, which is mediated by tyrosine kinase activity. Mechanistically, EGFR activation leads to downstream signaling through the BRAF/MAPK/ERK kinase (MEK) and PI3K/AKT/mTOR pathways, promoting oncogenic transformation and resistance to apoptosis [94]. EGFR overexpression is relatively common in CCA and has been reported to be associated with poor prognosis [95,96]. In both iCCA and eCCA, the 5-year overall survival rate was significantly lower in EGFR-positive patients than that in EGFR-negative patients, indicating that EGFR overexpression is an independent poor prognostic factor in CCA. However, the specific frequency of activating alterations in EGFR remains unclear, with two studies reporting rates of 13.6% [55] and 20% [56], respectively. These studies were small-scale, and it is difficult to discuss the results based on location.

PI3K encodes a lipid kinase that plays a central role in key intracellular signaling pathways linking receptor tyrosine kinases, G-protein-coupled receptors, and oncogenes to essential cellular functions, including proliferation, survival, and metabolism [97]. This pathway is tightly regulated by PI3K-generated phosphatidylinositol-3,4,5-trisphosphate (PtdIns(3,4,5)P_3_), whose levels are modulated by the tumor suppressor phosphatase and tensin homolog deleted on chromosome 10 (PTEN), a lipid phosphatase that dephosphorylates PtdIns(3,4,5)P_3_. Genetic alterations in PI3K, especially activating alterations in the p110α isoform (PIK3CA), are among the most frequent in various cancers. These alterations lead to the constitutive activation of the PI3K pathway, which contributes to oncogenesis. Inactivation or loss of PTEN results in hyperactivation of this pathway. Among these, PIK3CA, which encodes the p110α subunit, is known as a driver mutation in various cancers, including gastric cancer, breast cancer, ovarian cancer, and lung cancer [98,99]. According to recent reports, PIK3CA mutations are observed in 10–20% of advanced colorectal cancer cases, and the co-occurrence of alterations in APC, BRAF, EGFR, ERBB2, and KRAS is increasing [100]. These genomic instabilities have been reported to be associated with poor prognosis, highlighting the clinical significance of PIK3CA mutations. In CCA, the frequency of PIK3CA mutations is reported to be approximately 6% in iCCA and 3–7% in eCCA [48,57]. In a large-scale cell-free DNA analysis study in patients with advanced cholangiocarcinoma, the overall frequency of PI3CA mutations was reported to be 6.8% overall [101], confirming that this alteration is relatively rare in patients with CCA. However, as with other cancer types, further investigations are needed to clarify its role as a tumor driver gene.

BRAF belongs to the serine/threonine kinase family and encodes a serine/threonine kinase that plays a central role in the MAPK/ERK signaling pathway [102,103]. This pathway regulates key cellular processes such as proliferation, differentiation, and survival. Upon activation, BRAF phosphorylates its downstream targets, MEK and ERK, thereby transmitting signals to the nucleus and influencing gene expression. BRAF mutations, especially the well-known V600E mutation, in which valine is replaced by glutamic acid, lead to constant activation of the BRAF protein. This results in continuous stimulation of the MAPK signaling pathway and contributes to the development of cancer. V600E mutations are frequently reported in thyroid cancer [104] and melanoma [105], while non-V600E mutations are more common in colorectal cancer [106]. In CCA, the frequency of BRAF V600E mutations is relatively low, with 3–7% reported in iCCA, whereas nearly all eCCA cases are negative [4,58,59]. The diversity of BRAF mutations complicates cancer biology and poses treatment challenges. However, the discovery of BRAF mutations in various cancers has revolutionized the treatment approaches and led to the development of targeted therapies that inhibit BRAF-mediated signal transduction.

Breast cancer gene 1 (BRCA1) and BRCA2 are critical tumor suppressor genes whose protein products play essential roles in cellular DNA damage response. As key components of the homologous recombination repair pathway, they promote error-free repair of DNA double-strand breaks, thereby maintaining genomic stability [107]. Loss-of-function mutations in BRCA1/2 result in homologous recombination deficiency, leading to genomic instability and a significantly increased risk of developing cancers, particularly breast, ovarian, pancreatic, and prostate cancers. Tumors with BRCA1/2 mutations are highly sensitive to poly (ADP-ribose) polymerase (PARP) inhibitors and platinum-based chemotherapeutic agents via a mechanism of synthetic lethality and may exhibit reduced sensitivity to CDK4/6 inhibitors [108]. In CCA, the frequency of BRCA1/2 mutations in iCCA and eCCA has been reported to be approximately 3–5% [60,61]. Although their frequency is low, these alterations are considered to have clinical significance because of the potential usefulness of PARP inhibitors as a treatment option and the impaired DNA damage repair function, which suggests high sensitivity to DNA-crosslinking agents such as cisplatin.

SMAD4 is a central mediator of TGF-β signaling that regulates proliferation, differentiation, and apoptosis [109]. It acts as a tumor suppressor and is frequently mutated or deleted in pancreatic, colorectal, and CCA cancers [110,111]. According to an analysis based on next-generation sequencing in 75 patients with CCA, SMAD4 mutations were found in approximately 4% of iCCA cases and 25% of eCCA cases. SMAD4 mutations tend to be more common in eCCA and may be involved in tumor progression through impaired TGF-β-mediated proliferation inhibition and promotion of epithelial–mesenchymal transition [49]. The loss of SMAD4 protein expression in tissue specimens from patients with eCCA who underwent surgical resection has been shown to be associated with poor prognosis [112], supporting the clinical significance of SMAD4 mutations and deletions in CCA.

In addition to well-characterized genomic alterations such as KRAS and TP53, recent pan-cancer analyses have identified aberrant expression of RNA helicases as potential contributors to CCA biology. Notably, DEAD-box RNA helicase 1 (DDX1) has been reported to be highly expressed in CCA, with potential implications for prognosis and immune microenvironment modulation [113]. Although functional validation in CCA remains limited, such findings suggest that transcriptional regulators like DDX1 may complement established genomic markers in refining the molecular landscape of this cancer.

### 3.2. Genetic Abnormalities in CCA as Therapeutic Targets

IDH is an enzyme that converts isocitrate to α-ketoglutarate as part of the citric acid cycle and has three subtypes, IDH1, IDH2, and IDH3, depending on the site of action. IDH1 mutations are associated with malignant tumors, disrupt normal metabolic pathways, and lead to abnormal production of carcinogenic metabolites [114]. IDH mutations are genetic characteristics of CCA, glioblastoma, and acute myeloid leukemia. In CCA, IDH1 mutations are more common than IDH2 mutations, occurring in approximately 13–29% of iCCA cases and rarely observed in eCCA cases [53,62,63]. According to a retrospective study of patients with advanced iCCA who underwent chemotherapy, IDH1 mutations were observed in 14.5% of iCCA patients, and iCCA patients with IDH1 mutations appeared to have better tumor biological characteristics, including longer PFS, than those with IDH1 wild-type [115]. The presence of IDH1 mutations may confer increased sensitivity to conventional chemotherapy, and treatment methods that specifically inhibit mutant IDH1 are advancing toward clinical application. Therefore, IDH1 mutations in CCA do not necessarily correlate with a poor prognosis.

Abnormal regulation of FGFR signaling is known to cause cancer formation and progression, leading to treatment resistance, and FGFR gene abnormalities have been observed in various cancers. FGFR2 frequently forms fusions with partners such as BicC family RNA-binding protein 1 and transforming acidic coil–coil-containing protein 3 [20,116]. This gene functions as a major driver of carcinogenic chimeric proteins that cause tumor progression and activate abnormal signal transduction pathways. FGFR2 mutations, most of which involve gene fusion or rearrangements, are recognized as important therapeutic targets for CCA. FGFR2 mutations have been reported in 8–16% of iCCA and 0–2% of eCCA cases [20,53,64,65,66,67], and are one of the characteristic genetic alterations of iCCA. According to these reports, the positive rate of FGFR2 fusion in iCCA in Western countries is high, ranging from 10 to 20%. However, in a multicenter observational study in Japan, FGFR2 fusion was detected in 7–8% of ICC cases. Interestingly, there was variation in the positivity rate across anatomical locations of CCA and geographical regions. Recent large-scale population-based studies in Asia have reported that FGFR2 mutations in iCCA are significantly associated with HCV infection, female sex, and younger age [67]. Given the low frequency of FGFR2 mutations in Asia, active screening of these high-frequency subgroups may improve screening methods and enhance access to molecular targeted therapies.

ERBB2, also known as human epidermal growth factor receptor 2 (HER2), is a receptor tyrosine kinase belonging to the ERBB family with no known ligands. This protein is activated through homodimerization or heterodimerization with other activated ERBB receptors and induces downstream signal transduction via the MAPK and PI3K/AKT/mTOR pathways [117]. ERBB2 activation arises from gene amplification and overexpression and plays a well-established role in cancers such as breast, gastric, and endometrial carcinomas [118,119]. In these cancers, the tumor cells overexpress the ERBB2 receptor, resulting in enhanced ligand sensitivity and persistent activation of the MAPK and PI3K/AKT/mTOR pathways. Furthermore, ERBB2 gene alterations are characteristic genetic abnormalities in CCA, with frequencies reported to be 4–6% in iCCA and 3–20% in eCCA, showing a tendency to be more common in eCCA [48,68,69,70,71,72]. A recent retrospective study demonstrated that ERBB2 overexpression was associated with significantly shorter disease-free survival in patients with CCA who underwent curative resection than in those who did not, and it has been identified as an independent prognostic factor for recurrence after treatment [120]. However, similar to other cancers, ERBB2 mutations/amplifications appear to be poor prognostic factors for CCA. Nevertheless, molecular therapies targeting ERBB2 are increasingly being applied clinically, and further studies, including clinical trials for CCA, are anticipated.

### 3.3. Genomic Alterations According to Anatomical Subtypes of CCA

Genetic alterations in CCA are closely associated with histopathological subtypes defined by the WHO 2019 classification [121]. In particular, small-duct type iCCA arising in the context of chronic liver diseases, such as HBV or HCV infection, has been recognized as a distinct clinical and molecular entity, often exhibiting overlapping features with HCC. This subtype, which originates from peripheral bile ducts and is frequently observed in patients with chronic liver inflammation, is characterized by a higher frequency of BAP1 and IDH1/2 hotspot mutations and FGFR2 fusions [122,123], along with a lower incidence of KRAS mutations compared to the large-duct type. These molecular characteristics suggest a unique oncogenic pathway distinct from those of large-duct type iCCA or perihilar CCA, emphasizing the importance of taking underlying hepatic conditions into account when classifying iCCA molecularly [124].

In contrast, large-duct type iCCA, originating from larger intrahepatic bile ducts, is characterized by a higher prevalence of mutations in TP53, KRAS, and several TGF-β pathway-related genes, including SMAD4, TGFBR2, FBXW7, and MYC [125]. Furthermore, hotspot KRAS mutations have been shown to occur more frequently in periductal infiltrating-type CCA than in the mass-forming subtype [121], suggesting that the pattern of tumor growth may also be linked to specific molecular profiles. These findings highlight the importance of incorporating histopathological subtypes when interpreting the molecular landscape of CCA and developing targeted therapeutic strategies.

## 4. Genomic and Molecular Alterations by Etiology

### 4.1. CCA Associated with Chronic Hepatitis Viruses Infection

Although the exact frequency is often unknown, differences in genetic alterations associated with CCA by etiology have been reported, as shown in Table 2. In regions where HBV is prevalent, such as Asia, China, and South Korea, HBV infection is a risk factor for iCCA, and although not as strong as for iCCA, an association with eCCA has also been suggested [28]. In a cohort study of 103 patients with iCCA in China, potential driver genes, including TP53, IDH1, PTEN, and ARID1A, were identified, as previously reported [126]. Among these, TP53 is associated with a poor prognosis, and iCCA patients with TP53 mutations are more likely to be HBsAg-positive, suggesting that the p53-mediated pathway may contribute to tumorigenesis in HBV-induced iCCA, such as HCC [127]. In contrast, mutations in the KRAS oncogene are almost exclusively limited to patients with HBsAg-negative iCCA.

Chronic inflammation and fibrosis caused by HBV infection may increase the risk of CCA carcinogenesis, similar to that observed in HCC. In some cases, the distinction between iCCA and HCC is unclear, and differentiation from composite hepatocellular-cholangiocarcinoma may be difficult. HBV is not only detected in hepatocytes but also in bile duct epithelial cells, suggesting the possibility of direct viral effects. A recent study of 41 iCCA patients, including HBV-positive cases, showed that HBV DNA integration is frequently observed in iCCA tumors and combined hepatocellular cholangiocarcinoma, with recurrent insertions identified near oncogenes such as telomerase reverse transcriptase (TERT), mesenchymal–epithelial transition factor, and ALKBH5 [128]. These integration events are associated with large-scale genomic alterations and functional abnormalities in nearby cancer-related genes, suggesting that HBV-induced genetic instability plays a significant role in the molecular pathophysiology of CCA. Notably, HBV integration into the TERT promoter is frequent, mutually exclusive of TERT mutations, and is associated with promoter hyperactivation [129]. Other recurrent integration sites include the FAT atypical cadherin 2 gene, which activates genes regulating epithelial-mesenchymal transition, the mTOR pathway, and intergenic regions, suggesting that HBV contributes to tumor progression through multiple molecular pathways specific to iCCA.

On the other hand, although HCV infection is an important associated factor of CCA, comprehensive analysis of genetic alterations associated with HCV-related CCA appears to be limited at this time. According to a large-scale observational study in Japan involving 453 patients with CCA, the HCV antibody positivity rate was not high in 24 patients (5.3%). However, FGFR fusions or rearrangements were strongly associated with HCV antibody positivity, with an odds ratio of 9.50 [67].

**Table 2:** Genetic alterations in CCA by etiology.

Etiology	Subtype of CCA	Sample Size	Gene Alterations	Reference
HBV	iCCA	103 patients	TP53, IDH1, PTEN, and ARID1A	[126]
HBV	iCCA	41 patients	TERT	[128]
HCV	iCCA and perihilar CCA	24 patients	FGFR2	[67]
PSC	All	186 patients	TP53, KRAS, CDKN2A, SMAD4, and ERBB2	[130]
Liver Fluke	All	8 patients	TP53, KRAS, and SMAD4	[131]
Liver Fluke	All	108 patients	TP53	[132]
Liver Fluke	All	23 patients	ERBB2, TP53	[133]
Hepatolithiasis	iCCA	38 patients	KRAS	[134]

HBV: hepatitis B virus, HCV: hepatitis C virus, PSC: primary sclerosing cholangitis, iCCA: intrahepatic cholangiocarcinoma, TP53: tumor protein 53, IDH1: isocitrate dehydrogenase 1, PTEN: phosphatase and tensin homolog deleted on chromosome 10, ARID1A: AT-rich interactive domain 1A, TERT: telomerase reverse transcriptase, FGFR2: fibroblast growth factor receptor 2, KRAS: Kirsten rat sarcoma viral oncogene homolog, CDKN2A: cyclin-dependent kinase inhibitor 2A, SMAD4: SMAD family member 4, ERBB2: Erb-B2 receptor tyrosine kinase 2.

### 4.2. CCA Associated with Chronic Cholestatic Liver Diseases

Chronic cholestatic liver diseases—including PSC, PBC, hepatolithiasis, congenital hepatic fibrosis, liver fluke infection, and chemical exposure—share common pathogenic mechanisms involving chronic biliary inflammation and prolonged cholestasis, which collectively contribute to cholangiocarcinogenesis. Although the underlying etiologies differ, recent genomic and molecular profiling studies have revealed both shared and distinct alteration patterns depending on the cause.

PSC carries a high lifetime risk of CCA and represents a leading cause of mortality in affected patients [8]. Genomic analyses in a large-scale multicenter study of 186 patients revealed frequent typical alterations of eCCA, including TP53 (35.5%), KRAS (28.5%), CDKN2A (14.5%), and SMAD4 (11.3%), as well as potential therapeutic targets such as ERBB2 [130]. Sequential analyses of bile duct dysplasia demonstrated a progressive accumulation of alterations from low-grade dysplasia (e.g., FGFR1, CDKN2A, SMAD4, EGFR, and ERBB2) to high-grade dysplasia characterized by ERBB2 amplification (71%) and TP53 alterations (86%) [135]. These findings highlight the potential role of molecular profiling for early detection and the development of targeted therapies. Moreover, liquid biopsy using bile samples from PSC patients has shown promise, with promoter methylation of cysteine dioxygenase type 1, septin 9, vimentin, and others demonstrating high diagnostic accuracy for early CCA detection [136]. NGS of bile duct brush specimens also improves sensitivity for detecting high-grade dysplasia and early CCA beyond conventional cytology [137].

In PBC, the causal relationship with CCA remains less well defined, and data on associated genomic alterations are limited. Similarly, congenital hepatic fibrosis—characterized by chronic cholestasis and ductal plate malformation—has been sporadically associated with CCA, but large-scale molecular data are lacking.

CCA associated with Opisthorchis viverrini and Clonorchis sinensis infections is highly prevalent in Southeast Asia and exhibits a distinct molecular profile. Whole-exome sequencing in 8 patients, including O. viverrini–associated CCA, has revealed frequent mutations in TP53 (44.4%), KRAS (16.7%), and SMAD4 (16.7%) [131]. Comparative studies demonstrated significant geographic differences: TP53 mutations were more frequent in liver fluke-associated CCA, whereas BAP1 and IDH1/2 alterations predominated in non-fluke-related tumors [132]. A large-scale international study of 489 patients, including 23 liver fluke-positive CCA patients, confirmed recurrent ERBB2 amplifications and TP53 mutations in liver fluke-positive cases, while non-fluke-related CCA demonstrated more frequent FGFR2 rearrangements and epigenetic IDH1/2 and BAP1 alterations [133]. These findings underscore the importance of molecular profiling for guiding personalized therapy and highlight the potential of infection control for primary prevention, particularly in endemic regions.

Hepatolithiasis, common in East Asia, causes recurrent cholangitis and chronic bile stasis, predisposing to CCA through cycles of epithelial injury and regeneration. Similarly, chemical exposures, such as 1,2-dichloropropane, dichloromethane, and asbestos, have been linked to CCA development via mechanisms involving oxidative stress, DNA damage, and persistent biliary inflammation. The carcinogenesis of iCCA associated with hepatolithiasis is considered to progress stepwise from biliary intraepithelial neoplasia (BilIN), a precancerous lesion, to invasive CCA [134]. In a study involving patients with hepatolithiasis—including three without BilIN, 12 with low-grade dysplasia, 16 with high-grade dysplasia, 10 with carcinoma in situ, and 38 with iCCA—KRAS mutations were detected in 48% of BilIN cases and 31.5% of iCCA cases, while no KRAS mutations were observed in patients without BilIN [138]. Furthermore, the prevalence of KRAS mutations was highest in high-grade dysplasia, suggesting that this genetic alteration may occur at an early stage of the progression from BilIN to iCCA.

Despite diverse initiating factors, chronic cholestatic conditions converge on common oncogenic pathways, including TP53, KRAS, SMAD4, IDH1/2, and ERBB2 alterations. A unified understanding of these mechanisms provides opportunities for early molecular diagnosis, risk stratification, and targeted therapeutic strategies in CCA associated with chronic cholangiopathy.

### 4.3. CCA Associated with Other Liver Diseases

Regarding MASLD and ALD, which are potential precursors to HCC, cohort studies have suggested that they may increase the risk of developing CCA, but the number of actual cases is limited. Furthermore, these conditions often overlap with other risk factors, limiting the availability of evidence directly applicable to clinical practice. However, data on the genetic alterations associated with CCA progression are insufficient.

It should be noted that inconsistencies exist among published studies regarding both the epidemiological associations and the prevalence of genomic alterations. Such discrepancies may be attributable to differences in study design, cohort size, patient demographics, regional etiologies, and methodological approaches, and should be carefully considered when interpreting the available evidence.

## 5. Implications for Precision Therapy

Since the combination of gemcitabine and cisplatin has been shown to prolong OS in patients with unresectable CCA [18], it has remained the standard first-line treatment. More recently, regimens with immune checkpoint inhibitors have demonstrated some efficacy against CCA [139]; however, the benefits are limited compared to other cancer types, and the prognosis remains poor, particularly in patients with unresectable or metastatic disease. Against this backdrop, the identification of actionable genetic alterations has opened new avenues for molecularly targeted therapies. Representative molecular targets in CCA that have been clinically implemented to date are summarized in Table 3.

In clinical practice, targeted therapies such as FGFR2 and IDH1 inhibitors are typically positioned after failure of standard first-line chemotherapy or chemo-immunotherapy regimens. However, the optimal sequencing of these agents remains unresolved, as no prospective head-to-head studies have directly compared targeted therapy with immunotherapy-based combinations. Current evidence suggests that molecular profiling at diagnosis is essential to identify candidates for targeted therapies early, thereby enabling timely treatment decisions. Ongoing clinical trials are expected to provide further insights into sequencing strategies, including the potential benefits of integrating targeted inhibitors earlier in the treatment course or in combination approaches.

### 5.1. FGFR2 Mutation-Targeted Therapy

In recent years, clinical trials of multiple FGFR inhibitors targeting FGFR mutations in CCA have progressed, and this molecularly targeted therapy has emerged as one of the most significant treatments contributing to improved outcomes in patients with CCA. Pemigatinib was the first FGFR2 inhibitor approved by the Food and Drug Administration for the treatment of CCA with FGFR2 fusion/rearrangement positivity and is a selective and potent oral competitive inhibitor of FGFR1, FGFR2, and FGFR3 [140]. In the FIGHT-202 trial, the overall response rate (ORR) for pemigatinib was 35.5% in CCA patients with FGFR2 fusion or rearrangement, demonstrating superior efficacy compared to 0.0% in CCA patients without this genetic alteration. In patients with this genetic alteration, the median PFS following pemigatinib treatment was 6.9 months, and the median OS was 21.1 months [140]. Based on these positive results, pemigatinib is widely used in clinical practice in many countries.

Similarly, infigratinib, another selective FGFR1-3 inhibitor, has also been developed as a therapeutic agent for patients with FGFR mutations. In Phase II trials, infigratinib demonstrated an objective response rate of 23.1% in patients with FGFR2 fusion-positive CCA who had previously received gemcitabine-based therapy, suggesting its potential as an effective treatment [147]. However, in a subsequent Phase III trial, infigratinib failed to demonstrate superiority over the standard CG regimen in terms of both primary endpoints of PFS [141]. Infigratinib demonstrated a consistent efficacy of 37.9% ORR; however, owing to insufficient statistical power, definitive conclusions could not be drawn, highlighting the limitations of conducting clinical trials on the ultra-rare disease of FGFR2 mutation-positive cholangiocarcinoma.

Futibatinib, a next-generation FGFR inhibitor that inhibits FGFR1-4 through covalent binding, has demonstrated strong preclinical activity against acquired resistance alterations associated with antitumor activity and ATP-competitive FGFR inhibitors, making it the most recent and potentially effective drug available [148]. Futibatinib is an irreversible FGFR2 inhibitor that covalently binds to the C492 residue, providing extended suppression of FGFR activity and demonstrating superior efficacy against acquired resistance alterations compared with other FGFR inhibitors [149]. In a Phase II clinical trial, futibatinib demonstrated remarkable results in patients with FGFR2 fusion/rearrangement-positive iCCA, with an ORR of 42.0%, a median PFS of 9.0 months, and a median OS of 21.7 months [142]. Additionally, the treatment discontinuation rate in this clinical trial was 2%, indicating that infigratinib is effective regardless of treatment history or age, and offers superior maintenance of quality of life. On the basis of these results, fucitinib has been approved for use in several countries.

### 5.2. IDH1 Mutation-Targeted Therapy

Ivosidenib is a potent, orally administered, targeted IDH1 inhibitor originally developed for patients with IDH1-mutated acute myeloid leukemia [150]. In CCA, its efficacy was demonstrated in a Phase III placebo-controlled double-blind trial in patients with IDH1 mutation-positive cancer and a prior treatment history. In terms of treatment efficacy, the ORR for ivosidenib was 2%, with all responses being partial, and the disease control rate (DCR) was 53%. In contrast, the ORR for the placebo group was 0% and the DCR was 28%, suggesting that ivosidenib slowed disease progression [62]. The median PFS in the ibotenate group was 6.9 months, compared with 2.7 months in the placebo group, showing a significant difference. The final median OS in this trial was 10.3 months in the ivosidenib group, compared with 7.5 months in the placebo group, and the median OS in the placebo group after adjustment for crossover was 5.1 months [143]. Interestingly, despite the high crossover rate in this trial, ivosidenib demonstrated good tolerability and did not cause a decline in QOL, while showing a significant improvement in overall survival compared to the placebo, thereby demonstrating the clinical efficacy of ivosidenib in patients with advanced CCA harboring IDH1 mutations.

### 5.3. ERBB2 Mutation-Targeted Therapy

ERBB2 gene, also known as HER2 gene, is targeted by a combination of anti-HER2 antibody drugs, pertuzumab and trastuzumab, which have demonstrated significant improvements in treatment outcomes in patients with various types of cancer, including HER2-positive breast cancer and gastric cancer [151,152]. In a Phase IIa basket trial (MyPathway), 23% of patients with HER2-positive metastatic CCA achieved an ORR with trastuzumab plus pertuzumab. The treatment was generally well tolerated, with grade 3–4 treatment-emergent adverse events occurring in 46% of patients; however, no treatment-related grade 4 events or deaths were reported [144]. In an open-label Phase II trial involving 267 patients with HER2-positive tumors across seven tumor cohorts (endometrial cancer, cervical cancer, ovarian cancer, bladder cancer, CCA, pancreatic cancer, and others), ORR in all patients treated with the combination of anti-HER2 antibody and DNA topoisomerase I inhibitor, trastuzumab plus deruxtecan, was 37.1%, with a median PFS of 6.9 months and a median OS of 13.4 months [145]. In 41 patients with CCA, the ORR was 22.0%, the median PFS was 4.6 months, and the median OS was 7.0 months. Furthermore, while higher response rates are expected in strongly positive cases, there are many cases of interstitial pneumonia, and careful management of the side effects is necessary.

### 5.4. Therapies Targeting Other Alterations

Although BRAF mutations are less common in CCA than in melanoma or thyroid cancer, they activate the MAPK/ERK and MEK signaling pathways, promoting tumor proliferation and survival, and thus represent promising therapeutic targets. In the ROAR basket trial (a Phase II study), the efficacy of the RAF inhibitor dabrafenib combined with the MEK inhibitor trametinib was evaluated in 178 patients with BRAF V600E-mutated solid tumors, including those with refractory CCA [153]. Among 43 CCA patients, the ORR assessed by independent central review was 47%, indicating favorable efficacy [146]. Median PFS and OS were approximately 9 months and 14 months, respectively. Although grade ≥3 adverse events such as elevated γ-glutamyltransferase and fever were common, the treatment had a manageable safety profile.

The neurotrophic tropomyosin receptor kinase (NTRK) gene family encodes TRK receptors and includes three members: NTRK1, NTRK2, and NTRK3. Chimeric fusions involving these genes can serve as actionable targets in certain cancers. These fusions result in ligand-independent constitutive activation of TRK receptors, persistently activating oncogenic pathways such as MAPK, PI3K–AKT, and PLCγ, thereby driving uncontrolled cellular growth and survival [154]. Although rare in CCA overall (reported at ~0.2%), NTRK fusions have been detected in up to 3.6% of iCCA cases [155]. TRK inhibitors such as entrectinib and larotrectinib have demonstrated substantial antitumor activity across NTRK fusion-positive tumors. Entrectinib, which also inhibits ROS1, showed an ORR of 61.2%, median PFS of 13.8 months, and median OS of 33.8 months in a pooled analysis of 121 adult patients with NTRK fusion-positive tumors [156]. Notably, one patient with CCA achieved a partial response, with PFS of 12.0 months and OS of 23.4 months. Larotrectinib, a highly selective first-generation TRK inhibitor, has also shown robust and durable efficacy. In one clinical study involving 55 patients with NTRK fusion-positive tumors (including two with CCA), the ORR was 75%, with a median observation period of 9.9 months; at that time, median PFS had not been reached, and the 6- and 12-month PFS rates were 73% and 55%, respectively [157]. A recent updated analysis in TRK fusion-positive, treatment-naïve patients reported an ORR of 77%, with a median PFS of 59 months and median OS of 61 months [158]. Larotrectinib maintained consistent efficacy despite increased sample size and long follow-up.

## 6. Therapeutic Resistance and Future Direction in CCA

Despite recent advances in targeted therapies, CCA remains a highly lethal malignancy with limited durable responses. One major clinical challenge is the development of therapeutic resistance—both primary and acquired—driven by underlying molecular heterogeneity and adaptive oncogenic signaling.

Resistance to FGFR inhibitors in FGFR2-altered CCA occurs through multiple mechanisms, including on-target alterations and bypass signaling pathways [159]. Approximately 60% of patients develop secondary FGFR2 kinase domain mutations—most commonly at N550 and V565 residues—which limit the efficacy of reversible inhibitors but may remain sensitive to irreversible inhibitors such as futibatinib [149]. Additionally, bypass mechanisms, particularly involving MAPK pathway activations, contribute to acquired resistance. Some patients exhibit resistance without detectable genomic alterations, implicating adaptive signaling via wild-type EGFR or other ERBB family members. Preclinical models have shown that co-inhibition of EGFR enhances FGFR inhibitor responses, a strategy now under clinical investigation [160]. Furthermore, primary resistance may be associated with co-mutations such as TP53 alterations, which correlate with poor progression-free survival. Overall, the diversity of resistance mechanisms underscores the need for combinatorial approaches, longitudinal genomic monitoring, and transcriptomic profiling to optimize treatment strategies [64].

Similar to other molecular targeted therapies, tumors treated with mutant IDH inhibitors, such as ivosidenib, eventually develop resistance. While resistance mechanisms to FGFR inhibitors in CCA are well characterized, those related to IDH-targeted therapies remain poorly understood. Insights from acute myeloid leukemia suggest that receptor tyrosine kinase mutations, secondary IDH mutations, and isoform switching (e.g., IDH1 to IDH2) can mediate resistance [161]. In cholangiocarcinoma, a few clinical cases have reported similar resistance patterns, including secondary mutations that impair drug binding and acquired isoform switching [162]. However, such genetic mechanisms appear to be relatively rare, potentially explaining the limited tumor shrinkage observed with ivosidenib treatment in many patients.

Resistance mechanisms to HER2-targeted therapies have been most extensively studied in breast cancer, where key contributors include impaired binding to the HER2 extracellular domain, activating mutations in HER2, overexpression of alternative receptors such as EGFR, HER3, and VEGF, and downstream pathway activation via mutations in the PI3K/AKT/mTOR cascade [163]. In the clinical trial of neratinib for advanced biliary tract cancer, the mechanism of drug resistance was also investigated through paired analysis of tumor biopsy tissue and circulating cell-free DNA from patients who responded to treatment. This revealed the presence of acquired HER2 mutations [164]. In certain patients, disease progression was associated with a reduction in HER2 copy number and decreased variant allele frequency of the HER2 S310F mutation, implying a diminished reliance on HER2 signaling.

Resistance to dabrafenib and trametinib in BRAF-mutant melanoma has been widely investigated, revealing diverse mechanisms [165]. One key route involves activation of bypass pathways such as PI3K/AKT/mTOR, allowing tumor cells to maintain growth despite BRAF inhibition. Resistance is often heterogeneous but commonly involves reactivation of the MAPK signaling cascade [166]. Additionally, overexpression of receptor tyrosine kinases like EGFR and IGF-1R can also drive resistance through alternative downstream signaling [167].

Understanding and overcoming resistance to molecular targeted therapies in CCA remains a major clinical challenge. Future research should aim to further elucidate both genetic and non-genetic mechanisms of resistance, including epigenetic alterations, adaptive signaling, and tumor microenvironmental factors. In this context, the integration of liquid biopsy technologies, such as circulating tumor DNA and cell-free DNA analysis, offers a promising non-invasive approach for real-time monitoring of treatment response and the emergence of resistance alterations. These tools may facilitate early detection of resistance-associated alterations and enable timely therapeutic adjustments. Nevertheless, their clinical application in CCA is limited by variable sensitivity influenced by tumor burden, anatomical site, and technical factors, and should therefore be interpreted in conjunction with tissue-based analyses whenever feasible.

Looking ahead, exploratory studies, including those investigating natural compounds with cytotoxic and immunomodulatory effects in preclinical CCA models [168], highlight the potential for expanding the therapeutic landscape. While preliminary, such approaches underscore the importance of integrating innovative strategies with precision tools to achieve more individualized treatment paradigms.

## 7. Conclusions

Large-scale etiology-stratified genomic studies are essential for clarifying how chronic liver and biliary diseases contribute to the pathogenesis and molecular heterogeneity of CCA. While certain etiologies, such as PSC and liver fluke infection, exhibit distinct molecular signatures, many etiologic conditions, including HBV, HCV, MASLD, and ALD, remain insufficiently characterized at the genomic level. Integrating multi-omics data, including genomics, transcriptomics, epigenomics, and metabolomics, with real-world clinical outcomes and longitudinal patient cohorts is critical for uncovering clinically meaningful molecular subtypes. In parallel, advances in minimally invasive diagnostic platforms such as liquid biopsy and bile-based molecular testing offer promising tools for early detection and real-time disease monitoring. A deeper understanding of how etiologic factors influence tumor biology will not only refine personalized therapeutic approaches but also enhance risk stratification, surveillance, and preventive strategies tailored to region- and patient-specific contexts. Ultimately, such precision approaches hold the potential to significantly improve clinical outcomes in this challenging malignancy.

## Figures and Tables

**Table 1:** Functional roles and frequencies of gene alterations.

Gene Alterations	Functional Role	Frequency	References
KRAS	Transduces signals from cell surface receptors to downstream pathways to regulate cell proliferation and survival.	iCCA: 24–27% eCCA: 37–46%	[48,49,50,51,52]
TP53	Tumor suppressor regulating cell cycle arrest, DNA repair, apoptosis, and senescence in response to cellular stress	iCCA: 20–27% eCCA: 35–68%	[48,50,52,53]
ARID1A	Chromatin remodeling and transcription regulation; loss-of-function mutations contribute to tumorigenesis	iCCA: 18–23% eCCA: 14%	[48,50,53]
CDKN2A/B	Regulation of the G1/S cell cycle checkpoint via inhibition of CDK4/6	iCCA: 15–27% eCCA: 19%	[50,52,54]
EGFR	Regulation of cell proliferation, survival, and differentiation through tyrosine kinase signaling	All: 13.6–20%	[55,56]
PIK3CA	Driver of oncogenic PI3K signaling that promotes cell growth and survival	iCCA: 6% eCCA: 3–7%	[48,57]
BRAF^V600E^	Promotion of cell growth and survival through activation of the MAPK/ERK signaling pathway.	iCCA: 3–7% eCCA: nearly 0%	[4,58,59]
BRCA1/2	Maintenance of genomic stability through homologous recombination repair of DNA double-strand breaks	iCCA: 3% eCCA: 5%	[60,61]
SMAD4	Tumor suppressor mediating TGF-β signaling to regulate cell cycle arrest, apoptosis, and differentiation	iCCA: 4% eCCA: 25%	[49]
IDH1	Catalyzes the cytosolic step of the tricarboxylic acid cycle, converting isocitrate to α-ketoglutarate	iCCA: 13–29% eCCA: 0–5%	[53,62,63]
FGFR2	Oncogenic driver through aberrant activation of FGF signaling	iCCA: 8–16% eCCA: 0–2%	[20,53,64,65,66,67]
ERBB2	Promotion of cell proliferation and survival through activation of downstream MAPK and PI3K-AKT signaling pathways	iCCA: 4–6% eCCA: 3–20%	[48,68,69,70,71,72]

KRAS: Kirsten rat sarcoma viral oncogene homolog, iCCA: intrahepatic cholangiocarcinoma, eCCA: extrahepatic cholangioncarcinoma, TP53: tumor protein 53, ARD1A: AT-rich interactive domain 1A, CDKN2A/B: cyclin-dependent kinase inhibitor 2A/B, EGFR: epidermal growth factor receptor, PIK3CA: phosphatidylinositol-4,5-bisphosphate 3-kinase catalytic subunit alpha, PI3K: phosphoinositide 3-kinase, BRAF: B-Raf proto-oncogene, serine/threonine kinas, MAPK; mitogen-activated protein kinase, ERK: extracellular signal-regulated kinase, BRCA1/2: breast cancer gene 1/2, SMAD4: SMAD family member 4, TGF-β: transforming growth factor β, IDH1: isocitrate dehydrogenase 1, FGFR2: fibroblast growth factor receptor 2, ERBB2: Erb-B2 receptor tyrosine kinase 2, AKT: protein kinase B.

**Table 3:** Therapeutic targets and clinical development in CCA.

Target Alterations	Molecular Targeted Agents	Trial Phase	Clinical Trial Results	References
FGFR2 fusion/rearrangement	Pemigatinib	Phase II	ORR: 35.5%, PFS: 6.9 months, OS: 21.1months	[140]
FGFR2 fusion/rearrangement	Infigratinib	Phase III	ORR: 37.9%, PFS: 7.4 months, OS: NE	[141]
FGFR2 fusion/rearrangement	Futibatinib	Phase II	ORR: 42.0%, PFS: 9.0 months, OS: 21.7 months	[142]
IDH1 mutation	Ivosidenib	Phase III	ORR: 2.0%, PFS: 6.9 months, OS: 10.3 months	[62,143]
HER2 alteration	Pertuzumab plus Trastuzumab	Phase IIa	ORR:23%	[144]
HER2 alteration	Trastuzumab plus Deruxtecan	Phase II	ORR: 37.1%, PFS: 6.9 months, OS: 13.4 months	[145]
BRAF^V600E^ mutation	Dabrafenib plus Trametinib	Phase II	ORR: about 47%, PFS: about 9 months, OS: about 14 months	[146]

FGFR2: fibroblast growth factor receptor 2, ORR: overall response rate, IDH1: isocitrate dehydrogenase 1, HER2: human epidermal growth factor receptor 2, BRAF: B-Raf proto-oncogene, serine/threonine kinase.

## Data Availability

No new data were created or analyzed in this study. Data sharing is not applicable to this article.

## Abbreviations

AKT	Protein kinase B
ALD	Alcohol-related liver disease
ALK	Anaplastic lymphoma kinase
ARD1A	AT-rich interactive domain 1A
BAP1	BRCA1-associated protein 1
BillN	Biliary intraepithelial neoplasia
BRAF	B-Raf proto-oncogene, serine/threonine kinase
BRCA1/2	Breast cancer gene 1/2
CCA	Cholangiocarcinoma
CDKN2A/B	Cyclin-dependent kinase inhibitor 2A/B
DCR	Disease control rate
DDX1	DEAD-box RNA helicase 1
EGFR	Epidermal growth factor receptor
ERBB2	Erb-B2 receptor tyrosine kinase 2
ERK	Extracellular signal-regulated kinase
eCCA	Extrahepatic cholangiocarcinoma
FGFR2	Fibroblast growth factor receptor 2
HBV	Hepatitis B virus
HCC	Hepatocellular carcinoma
HCV	Hepatitis C virus
HER2	Human epidermal growth factor receptor 2
iCCA	Intrahepatic cholangiocarcinoma
IDH1	Isocitrate dehydrogenase 1
KRAS	Kirsten rat sarcoma viral oncogene homolog
MAPK	Mitogen-activated protein kinase
MASLD	Metabolic dysfunction-associated steatotic liver disease
MEK	MAPK/ERK kinase
mTOR	Mechanistic target of rapamycin
NGS	Next-generation sequencing
NTRK	Neurotrophic tyrosine receptor kinase
ORR	Overall survival rate
OS	Overall survival
PARP	Poly ADP-ribose polymerase
PBC	Primary biliary cholangitis
PFS	Progression-free survival
PI3K	Phosphoinositide 3-kinase
PIK3CA	Phosphatidylin-ositol-4,5-bisphosphate 3-kinase catalytic subunit alpha
PSC	Primary sclerosing cholangitis
PTEN	Phosphatase and tensin homolog deleted on chromosome 10
PtdIns(3,4,5)P_3_	PI3K-generated phosphatidylinositol-3,4,5-trisphosphate
RFS	Recurrence-free survival
SMAD4	SMAD family member 4
SVR	Sustained virological response
TERT	Telomerase reverse transcriptase
TGF-β	Transforming growth factor β
TP53	Tumor protein 53
TRK	Tropomyosin receptor kinase
